# Effects of electrical stimulation combined with strength training on pain, muscle strength and lower-limb biomechanics in patellofemoral pain patients: a randomized controlled trial

**DOI:** 10.1186/s12891-025-09465-3

**Published:** 2025-12-30

**Authors:** Xiaowei Yang, Boshi Xue, Dong Sun, Minjun Liang, Yingce Yao, Chen Yang, Zhipeng Zhou, Yaodong Gu, Peimin Yu, Liangliang Xiang

**Affiliations:** 1 Faculty of Sports Science, Ningbo University, Ningbo, 315211 China; 2 Faculty of Health Sciences and Sports, Macao Polytechnic University, Macao, 999078 China; 3 Qilu Hospital of Shandong University Dezhou Hospital, Dezhou, 253000 China; 4 School of Sports and Health, Nanjing Sport Institute, Nanjing, 210014 China; 5 College of Sports and Health, Shandong Sport University, Jinan, 250102 China; 6 Department of Engineering Mechanics, KTH MoveAbility, KTH Royal Institute of Technology, Stockholm, 10044 Sweden

**Keywords:** Electrical stimulation, Patellofemoral joint force, Strength training, Movement patterns, Muscle activation

## Abstract

**Background:**

Insufficient vastus medialis (VM) activation and excessive patellofemoral joint loading are primary contributors to patellofemoral pain (PFP). Although electrical muscle stimulation can reduce pain and enhance strength, evidence for its efficacy in selectively strengthening the VM and improving lower-limb biomechanics in PFP patients remains limited. This study aimed to investigate the clinical efficacy of electrical stimulation combined with strength training versus conventional strength training in PFP patients.

**Methods:**

Forty-six participants were randomly assigned to an electrical muscle stimulation combined strength training (EMS) group and a muscle strength training (MST) group. Both groups completed a 6-week hip/knee strengthening program (3 sessions/week, 60 min/session) whereas the EMS group received the extra electrical stimulation of VM during the knee training. Prior to and after the intervention, participants performed stair descent and isokinetic strength tests. Subjective knee pain and functional capacity were assessed using patient-reported measures, while kinematic, kinetic, and surface electromyography data were collected during stair descent.

**Results:**

After the 6-week intervention, both groups showed reduced knee valgus angle, hip internal rotation angle, patellofemoral joint stress and reaction force, along with decreased activation of the gluteus medius, gluteus maximus, and vastus lateralis and increased VM activation during stair descent (all *p* < 0.05). In addition, both groups exhibited increased hip and knee muscle strength (all *p* < 0.05). Compared with the MST group, the EMS group demonstrated greater improvements in medial-lateral quadriceps activation ratio and knee extension strength, alongside more reductions in knee external rotation angle, hip adduction angle, and the anterior knee pain scale scores (all *p* < 0.05).

**Conclusions:**

EMS combined with strength training more effectively mitigated abnormal hip–knee movement patterns during stair descent, balanced the activation of the vastus medialis and lateralis, increased knee extensor strength, and alleviated pain and enhanced function in PFP patients.

**Trial registration:**

Trial Registration Number: ChiCTR2300067598, Date of trial registration: 1/13/2023.

**Supplementary Information:**

The online version contains supplementary material available at 10.1186/s12891-025-09465-3.

## Background

Patellofemoral pain (PFP) is a complex multifactorial condition [1], marked by diffuse peri- and/or retro-patellar pain during weight-bearing knee flexion such as stair walking, squatting, and jump landing [2]. Its prevalence is up to 22.7% in the general population, particularly common among adolescents and adults [3]. Moreover, nearly 80% of PFP cases present recurrent or chronic symptoms [4], potentially progressing to irreversible knee osteoarthritis [5].

Excessive patellofemoral joint (PFJ) loading is considered a potential contributing factor in the development of PFP, commonly arising from impaired lower extremity mechanics such as reduced activation of the vastus medialis (VM) and weakness of the gluteal muscle [6–8]. The quadriceps play a crucial role in maintaining normal patellar tracking, and imbalance between medial and lateral quadriceps strength may result in lateral patellar displacement and accelerated cartilage degeneration [9, 10]. For example, PFP patients exhibit reduced and delayed VM activation during running, a change that has been confirmed in vitro biomechanical studies to increase patellofemoral joint loading and lateral patellar displacement [11–13]. Furthermore, weakness in hip abductors and external rotators may induce excessive femoral adduction or internal rotation under load, thereby exacerbating lateral patellar forces and pain [14, 15]. Research indicates that females with PFP exhibit greater peak hip internal rotation compared to healthy controls during activities including running, stair descent, and landing [16]. Therefore, prevention and rehabilitation for PFP should focus on targeted training of the VM and strengthening of hip abductors and external rotators to reduce the patellofemoral joint loading.

Strength training of the muscles around the knee and hip is the cornerstone of PFP rehabilitation, but its efficacy remains uncertain and patient outcomes are often suboptimal [17]. Blønd et al. reported that, following a PFP rehabilitation program, 80% of patients continued to experience pain, with 74% reducing their physical activity levels over a 5-year follow-up period [18]. This may be related to the fact that traditional strength training aimed at selectively increasing VM activation does not produce additional VM activation or improvements in PFP symptoms [19, 20]. Drawing on the anatomical properties of the VM, several studies have sought to preferentially recruit this muscle by concurrently activating knee extensors, hip adductors, or inducing active tibial internal rotation [21, 22]. However, these attempts have largely failed, as research has not demonstrated the intended preferential recruitment. This indicates that isolated recruitment of the VM via lower-limb strength training may be inadequate. Consequently, rehabilitation for patients with PFP may necessitate the incorporation of innovative intervention strategies to precisely activate and strengthen the VM.

Electrical muscle stimulation (EMS) represents a promising adjunct that facilitates neuromuscular re-education, and is widely utilized to enhance muscle strength and alleviate pain [23]. Research demonstrates that incorporating EMS into early rehabilitation for patients undergoing anterior cruciate ligament (ACL) reconstruction can improve muscle strength and reduce pain relative to conventional physical therapy alone [23, 24]. EMS can significantly increase the cross-sectional area of VM fibers and enhance strength in patients with PFP [25]. Nevertheless, its effects on hip and knee muscle activation, patellofemoral joint loading, and aberrant biomechanics during functional tasks remain uncertain. Furthermore, stair descent is one of the most common symptom-provoking activities in patients with PFP and places greater demands on eccentric quadriceps control and dynamic knee stabilization than level walking [26, 27], with patellofemoral joint loading estimated to be approximately 2 times higher than during level-ground gait [28]. However, biomechanical studies specifically examining stair descent in PFP patients remain relatively limited. Accordingly, assessing lower-limb biomechanics during stair descent provides a clinically relevant and mechanically demanding task for evaluating the effects of EMS combined with strength training.

Consequently, this study was aimed to explore the effects of a 6-week intervention combining EMS and strength training on knee pain, lower-limb muscle strength, and hip/knee joint biomechanics and muscle activation during stair descent in patients with PFP. It was hypothesized that, post-intervention, the EMS group would demonstrate reduced patellofemoral joint loading and visual analog scale (VAS) scores, increased knee extension strength and anterior knee pain scale (AKPS) scores, elevated VM activity and activation ratio between VM and vastus lateralis (VL), and modified movement patterns (characterized by decreased knee abduction and external rotation, as well as reduced hip adduction and internal rotation) relative to the MST group.

## Methods

### Study design

**Fig. 1:**
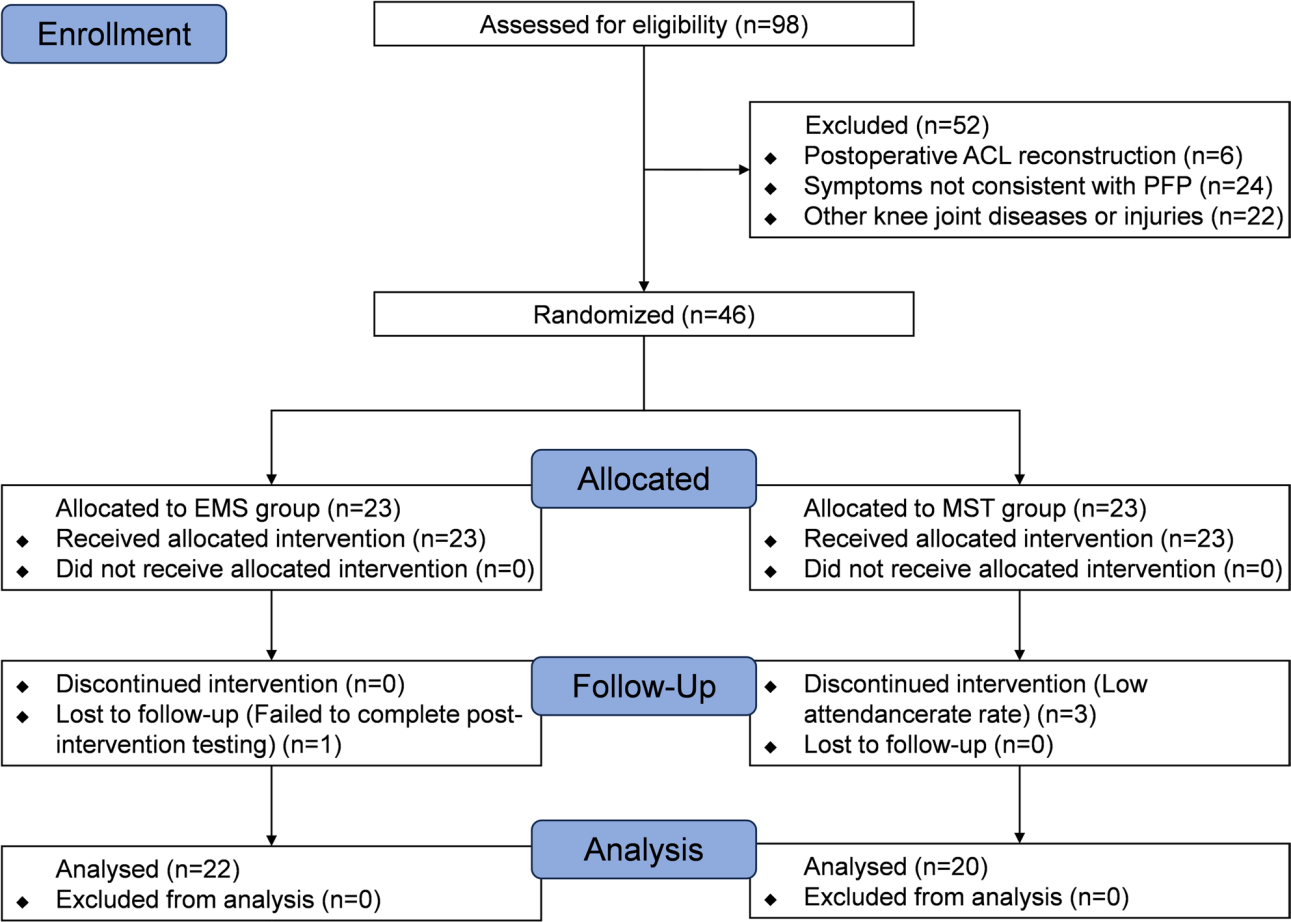
Flow chart

This study was designed and conducted in accordance with the CONSORT guidelines for randomized controlled trials. It was a single-blind, randomized controlled trial with parallel groups of patients with PFP. The flowchart of the study design is presented in Fig. 1.

### Randomization

A computer-generated randomization sequence with a block size of 6 was created using SPSS 27.0 (IBM Corp., Armonk, NY, USA), and based on this sequence, participants were assigned to either the EMS or MST group.

### Allocation concealment

Sealed, consecutively numbered envelopes prepared by independent researchers were used to ensure allocation concealment and minimize selection bias. These envelopes remained unopened until each participant finished baseline evaluations, ensuring recruiters and therapists remained blind to assignment.

### Blinding

To ensure objectivity in both assessments and intervention procedures, a single designated researcher responsible for clinical evaluations and the experienced physical therapists who administered the interventions were blinded to group allocation.

The study was approved by the Ethics Committee of Sports Science at Shandong Sports University (Approval No. 2022012) in accordance with the Declaration of Helsinki (https://www.wma.net/policies-post/wma-declaration-of-helsinki/) and registered with the Chinese Clinical Trial Registry (No. ChiCTR2300067598). Prior to the study, all participants were fully informed of the study procedure and provided informed consent. Participants retain the right to unconditionally withdraw from this study.

### Participants

Effect sizes derived from previous studies were d = 0.77 for VAS (time effect) [29], f = 0.48 for patellofemoral joint reaction force (time × group interaction) [30], and d = 1.02 for VM activation (group effect) [31]. A priori power analyses (G*Power Version 3.1) using these effect sizes with a type I error level of 0.05 indicated that sample sizes of 16, 12, and 17 in each group, respectively, are required to achieve 80% statistical power. Based on a 20% anticipated drop-out rate and to obtain a more robust power estimate, a total of 46 participants were ultimately enrolled.

All PFP patients were recruited between February 2023 and October 2023 from Shandong Sport University, Jinan, China. They were assessed by the same experienced professional rehabilitation therapist, and their medical histories were reviewed in detail. The inclusion criteria were based on previous studies, including [32, 33]: (i) Aged 18–40 years; (ii) Non-traumatic knee pain lasting more than 3 months; (iii) Unilateral anterior or posterior patellar pain during at least two of the following activities: running, squatting, jumping, prolonged sitting or kneeling, stair climbing, and isometric contraction of the quadriceps; (iv) VAS score ≥ 3; (v) Willingness to accept and consent to the use of low-frequency electrical stimulation. The exclusion criteria were as follows: (i) Presence of ACL tear, meniscal injury, osteoarthritis, patellar tendinitis, etc.; (ii) Patellar dislocation (including subluxation); (iii) History of previous surgery on the lower limbs or trunk; (iv) Inability to tolerate electrical muscle stimulation or presence of contraindications to electrical stimulation therapy; (v) Receipt of other rehabilitation treatments within the past three months.

### Protocol

All participants completed a 6-week strength training program, with the EMS group incorporating electrical muscle stimulation, as detailed in the Intervention Program section below. Stair descent tests and isokinetic strength tests of the knee and hip joints were performed pre- and post-training for both groups, during which VAS and AKPS data for the affected knee were collected. Both the intervention program and testing procedure were under the guidance of experienced physical therapists and specialized researchers. Attendance records and exercise diaries were used to monitor adherence to the intervention, in which participants recorded their exercise activities and any issues encountered. If participants experience intolerable pain or any serious discomfort, the intervention would be terminated immediately, and medical care would be provided as necessary.

### Intervention program

Both the EMS and MST groups participated in a 6-week hip and knee strength training program, administered three times weekly for 60 min per session [23, 34]. The intervention program schedule is presented in Table 1. In addition to the core strength training, warm-up and stretching exercises were incorporated [34, 35]. To mitigate the effects of fatigue on testing, a 2–3 min rest was mandated between each training set, with a minimum of 48 h between sessions. Throughout the training program, instructors delivered visual, tactile, and verbal feedback to rectify participants’ body movements.

**Table 1:** Six-week intervention program for the EMS and MST groups

	Set × repetitions or seconds × load					
	Week 1	Week 2	Week 3	Week 4	Week 5	Week 6
Knee extension with resistance*	3 × 15 × 70% 1RM	3 × 15 × 70% 1RM	3 × 15 × 70% 1RM	3 × 15 × 70% 1RM	3 × 15 × 70% 1RM	3 × 15 × 70% 1RM
Wall squat hold	3 × 60 s	3 × 60 s	3 × 90 s	3 × 90 s	3 × 120 s	3 × 120 s
Clamshell hip abduction	3 × 15 × 10 LB	3 × 15 × 10 LB	3 × 15 × 15 LB	3 × 15 × 15 LB	3 × 15 × 20 LB	3 × 15 × 20 LB
Band-assisted glute bridge	3 × 15 × 10 LB	3 × 15 × 15 LB	3 × 15 × 20 LB	-	-	-
Bodyweight squat*	3 × 15	3 × 15	3 × 15	-	-	-
Hip thrust with barbell	-	-	-	3 × 15 × 70% 1RM	3 × 15 × 70% 1RM	3 × 15 × 70% 1RM
Smith machine squat*	-	-	-	3 × 15 × 70% 1RM	3 × 15 × 70% 1RM	3 × 15 × 70% 1RM

The strength training program for both groups consisted of 7 exercises, with an intervention period of 6 weeks. RM, repetition maximum; LB, pound

*Denotes that the EMS group received electrical stimulation while performing this exercise

**Fig. 2:**
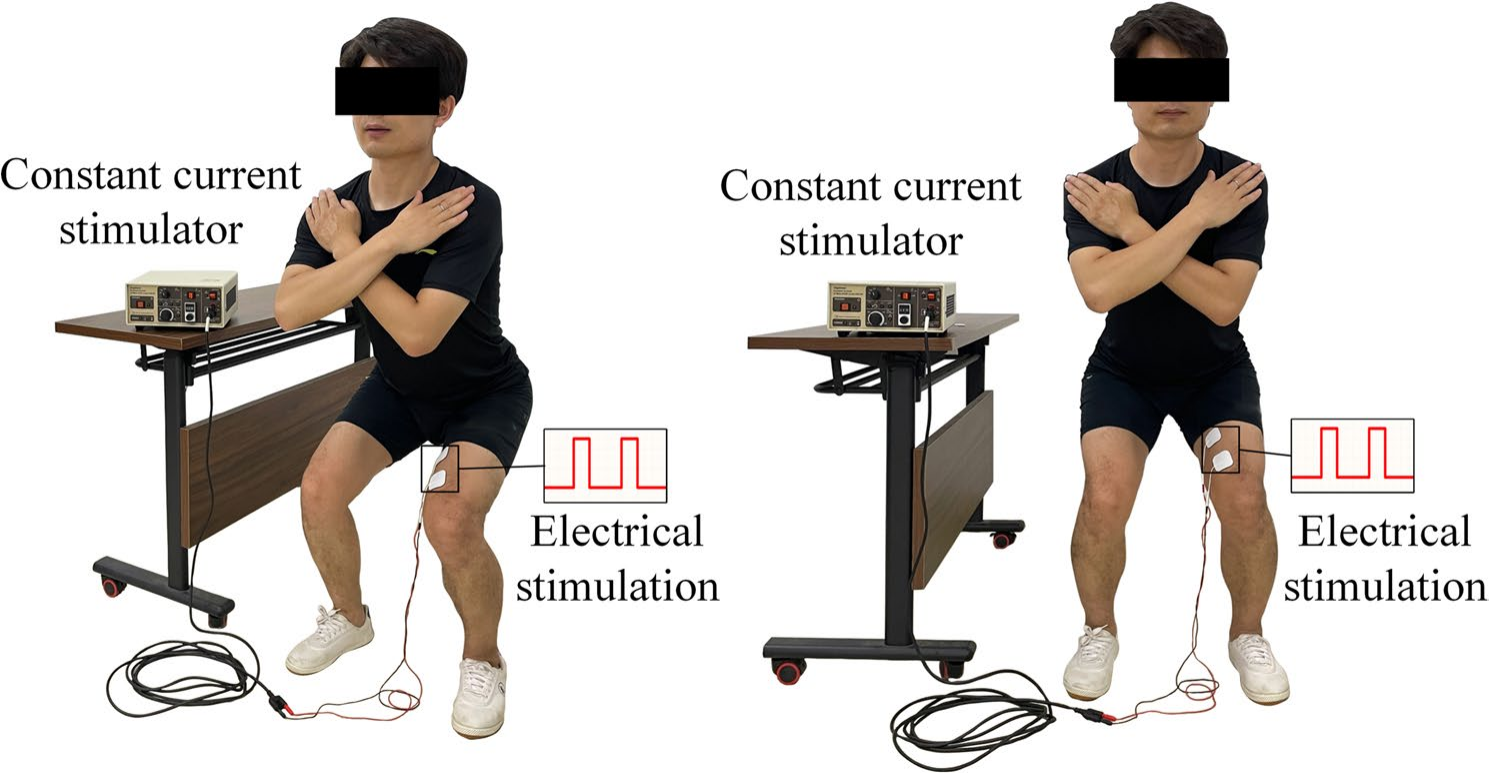
EMS group received electrical muscle stimulation during knee strength training (left: lateral view; right: anterior view)

As discussed above, the balance between VM and VL plays a crucial role in maintaining lateral patellar stability during knee movement, thereby reducing stress on the patellofemoral joint. In addition, considering participant acceptability and the safety of the training protocol, we applied additional VM-targeted electrical stimulation in only three of the seven training exercises. In the EMS group, self-adhesive electrode pads (40 × 40 mm) were positioned along the VM fibers of the affected leg, with the cathode located 4 cm superior and 3 cm medial to the patella, and the anode positioned 10 cm superior to the patella along the medial thigh line [23]. As shown in Fig. 2, during knee joint strength training, the EMS group underwent VM stimulation via a constant current stimulator (Digitimer DS7A, UK) configured to deliver a square wave (pulse width: 400 µs, frequency: 50 Hz) [23, 36]. The current intensity was individualized to each participant to elicit maximal VM contraction while minimizing discomfort [23, 36]. To account for increasing tolerance, the current intensity was re-adjusted weekly according to individual variations.

### Data collection

#### Stair descent test

**Fig. 3:**
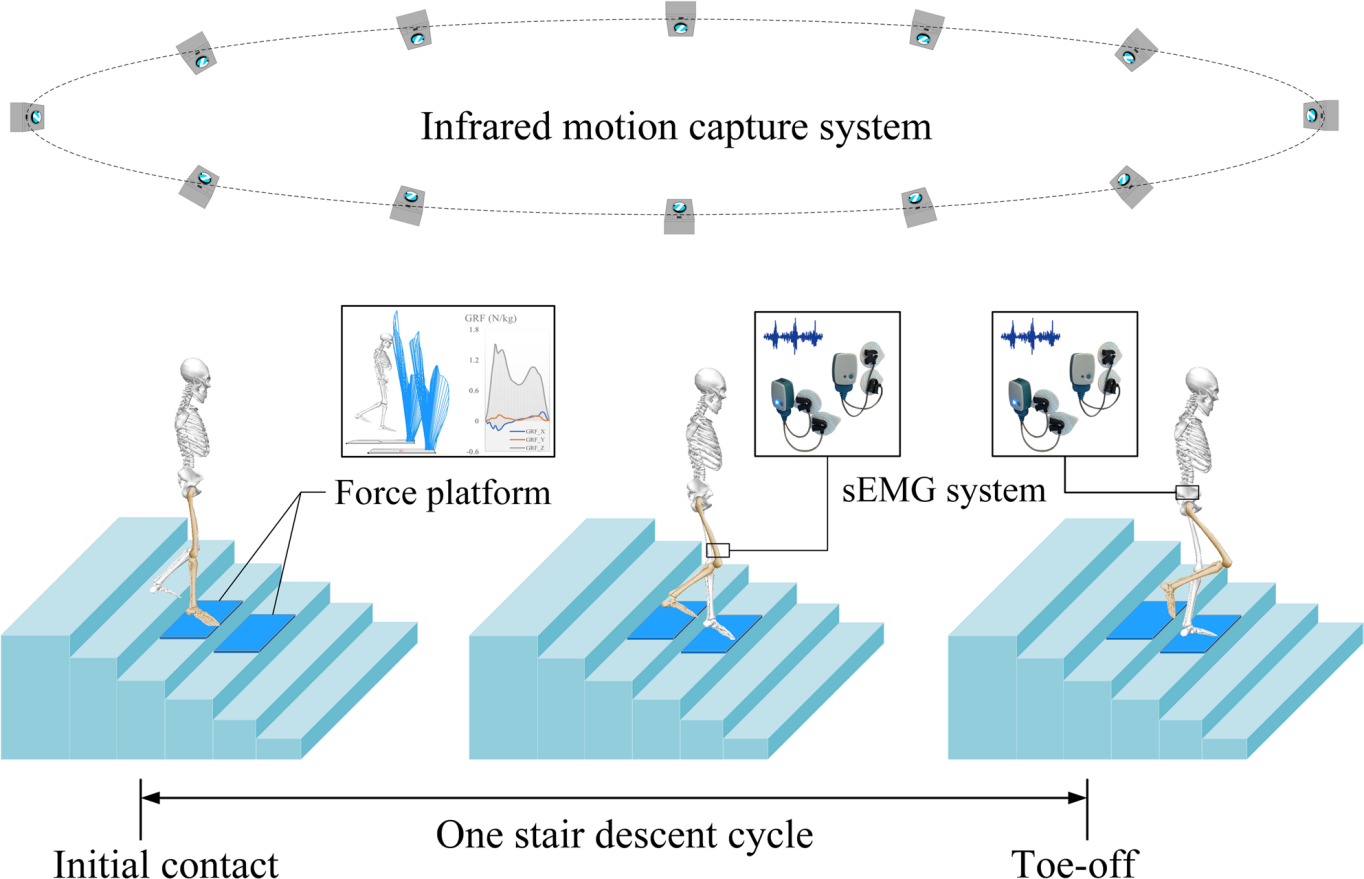
Stair descent schematic. One stair descent cycle is the phase from initial contact with the force platform to toe-off of the affected limb; sEMG, surface electromyography

During the stair descent test, participants wore tight-fitting clothing and standardized athletic shoes. Following a warm-up period, surface electromyography (EMG) electrodes were placed on the vastus medialis/lateralis and gluteus maximus/medius muscles of the affected leg [11, 37]. Forty-one reflective markers were placed on the head (*n* = 4), trunk (*n* = 4), pelvis (*n* = 5), knee joints (*n* = 4), ankle joints (*n* = 4), and feet (*n* = 8) according to the Helen Hayes marker set, with three marker clusters on each thigh (*n* = 6) and shank (*n* = 6) [38]. All participants were instructed to complete three successful stair descent trials. Participants were directed to descend at a self-selected, comfortable pace. A “one-step-per-stair” approach was mandated, prohibiting skipped steps to accurately replicate daily stair ambulation patterns [39] (Fig. 3). EMG activity of muscles in the affected limb was normalized using maximum voluntary isometric contraction (MVIC) [26]. The MVIC tests for VM and VL were performed with participants seated and the knee flexed to 60°. The MVIC tests for gluteus maximus and gluteus medius were performed in the prone and side-lying positions, respectively, with the knee fully extended. Each test consisted of three 5-s maximal contractions, with a 2-minute rest period between trials.

In this study, raw three-dimensional marker coordinates were collected via 12 infrared cameras (100 Hz, Vicon Motion Systems Ltd., Oxford, UK). Ground reaction force (GRF) data for affected limb were recorded using an AMTI force platform (1000 Hz, AMTI, Inc., Watertown, MA, US). Surface EMG signals for affected leg were synchronously collected using four NORAXON surface EMG sensors (2000 Hz, Noraxon, Arizona, US).

#### Isokinetic strength test

Isokinetic testing was performed using an IsoMed 2000 dynamometer (D&R Ferstl GmbH, Hemau, Germany) at an angular velocity of 60°/s. After a 5-minute general warm-up, participants were positioned according to the manufacturer’s recommendations. Knee flexion-extension strength testing was conducted in the seated position with the hip flexed to 85°. Participants performed maximal voluntary contractions through the full range of motion. Hip strength testing was performed in both the supine and side-lying positions. Maximal voluntary contractions were carried out for extension, external rotation, and abduction across the full range of motion. For each degree of freedom, three sets of five repetitions were completed, with a 2-minute rest interval between sets.

#### Patient-reported outcome measures

Pain intensity and functional capacity were assessed using the VAS and AKPS. To evaluate pain, participants performed a single-leg squat test to provoke knee discomfort, after which they rated their subjective pain on the VAS, ranging from 0 (no pain) to 10 (worst pain imaginable) [40, 41]. Functional limitations were further examined using the AKPS, a 13-item questionnaire with scores ranging from 100 (no impairment) to 0 (severe impairment). Lower scores on the AKPS indicate greater pain severity and functional impairment [42].

### Data reduction

A stair descent cycle was defined as the interval from heel contact of the affected limb (vertical GRF ≥ 10 N) to toe-off (vertical GRF < 10 N) [43]. A Butterworth low-pass filter with a cutoff frequency of 10 Hz was applied to smooth the raw coordinate signals of each marker [44]. GRFs were filtered using a low-pass filter with a cutoff frequency of 50 Hz [44]. Joint angles were computed based on the relative orientation between the distal and proximal segments, employing a Cardan rotation sequence (X-Y-Z) [45]. Joint net moments (Nm) were determined using an inverse dynamics algorithm [46] and normalized to body weight (kg) as Nm·kg⁻¹. Flexion, adduction, and internal rotation were assigned positive values, whereas extension, abduction, and external rotation were assigned negative values for joint angles and moments. Patellofemoral joint stress was computed using knee joint angle and moment, then normalized to body weight (kg), and expressed in units of MPa·kg⁻¹ [47].

The calculation formula is as follows:

1 $$\:{CA}_{\mathrm{P}\mathrm{F}\mathrm{J}}=2.0{\mathrm{e}}^{-5}{\alpha\:}_{\mathrm{knee}}^{4}-0.0033{\alpha\:}_{\mathrm{knee}}^{3}+0.1099{\alpha\:}_{\mathrm{knee}}^{2}+3.5273{\alpha\:}_{\mathrm{knee}}+81.058$$

2 $$\:{L}_{\mathrm{q}}=8.0{\mathrm{e}}^{-5}{\alpha\:}_{\mathrm{knee}}^{3}-0.013{\alpha\:}_{\mathrm{knee}}^{2}+0.28{\alpha\:}_{\mathrm{knee}}+0.6$$

3 $$\:{F}_{\mathrm{q}}=\frac{{M}_{\mathrm{knee}}}{{L}_{\mathrm{q}}}$$

4 $$\:k=\frac{-3.8{\mathrm{e}}^{-5}{\alpha\:}_{\mathrm{knee}}^{2}+1.5{\mathrm{e}}^{-3}{\alpha\:}_{\mathrm{knee}}+0.462}{-7.0{\mathrm{e}}^{-7}{\alpha\:}_{\mathrm{knee}}^{3}+1.6{\mathrm{e}}^{-4}{\alpha\:}_{\mathrm{knee}}^{2}+0.016{\alpha\:}_{\mathrm{knee}}+1}$$

5 $$\:{F}_{\mathrm{P}\mathrm{F}\mathrm{J}}=k\times\:{F}_{\mathrm{q}}$$

6 $$\:PFJ\:stress=\frac{{F}_{\mathrm{P}\mathrm{F}\mathrm{J}}}{{CA}_{\mathrm{P}\mathrm{F}\mathrm{J}}}$$

Where *CA*_PFJ_ represents the contact area of the patellofemoral joint, *L*_q_ is the equivalent moment arm of the knee joint, *F*_q_ is the quadriceps knee extension force, *k* is a constant, and F_PFJ_ is the patellofemoral joint reaction force.

A band-pass filter (20–500 Hz) was applied to raw EMG signals, followed by full-wave rectification [48]. Subsequently, a low-pass filter with a cutoff frequency of 20 Hz was used for smoothing the signals, thereby attenuating low-frequency noise. Muscle activation during stair descent was normalized using the average EMG amplitude recorded over the middle 3 s of three MVIC trials. Peak torques from isokinetic tests were measured to evaluate muscle strength, calculated as the maximum value of three tests (Nm) normalized to body weight (kg), and expressed as relative peak torque (Nm·kg⁻¹).

### Statistical analysis

The per-protocol approach was applied to analyze the data, including only participants who fully adhered to the trial protocol and completed both the intervention and follow-up. The Shapiro-Wilk test was used to assess the normality of all data. Two-way mixed-design ANOVAs were conducted to assess the main effects of time (week 0 and week 7) and group (EMS and MST), along with their interaction, for each dependent variable. If a significant interaction effect was detected, simple-effects analyses were performed to interpret the interaction, with p-values adjusted for multiple comparisons using the Bonferroni method. The effect size was calculated using partial η^2^ (η2 *p*), where 0.01 ≤ η2 *p* < 0.06 indicates a small effect, 0.06 ≤ η2 *p* < 0.14 indicates a medium effect, and η2 *p* ≥ 0.14 indicates a large effect [49]. All data were presented as mean ± standard deviation (M ± SD) and were statistically analyzed in SPSS 27.0 (IBM Corp., Armonk, NY, USA). An alpha level of 0.05 was applied for all statistical tests.

## Results

A total of 46 participants were randomly assigned to either the EMS group (*n* = 23) or the MST group (*n* = 23). One participant in the EMS group failed to complete the post-intervention test due to loss of contact, while three participants in the MST group were excluded because personal scheduling conflicts prevented them from attending the required number of intervention sessions. All other participants completed the intervention and testing under the guidance of physical therapists and researchers, strictly adhering to the study protocol. They maintained their usual lifestyles throughout the study period but received no other treatments. Ultimately, outcomes from 22 participants in the EMS group and 20 participants in the MST group were included in the analysis. No serious adverse reactions, such as intolerance to electrical stimulation, were reported by any participants during the trial. The basic information of the participants is shown in Table 2.

**Table 2:** Participant information

	EMS group (*n* = 22)	MST group (*n* = 20)	*P*
Age (years)	21.3 ± 3.6	20.5 ± 2.1	0.765
Height (cm)	173.8 ± 8.1	174.7 ± 8.4	0.695
Weight (kg)	73.2 ± 13.3	69.5 ± 11.7	0.342
Gender (male/female, n)	17/5	13/7	0.392
Affected side (left/right, n)	9/13	8/12	0.954
VAS score	4.7 ± 0.7	4.6 ± 0.9	0.598
AKPS score	77.0 ± 7.3	75.7 ± 5.3	0.522

EMS, electrical muscle stimulation; MST, muscle strength training; VAS, visual analog scale; AKPS, anterior knee pain scale

### AKPS and VAS scores

**Fig. 4:**
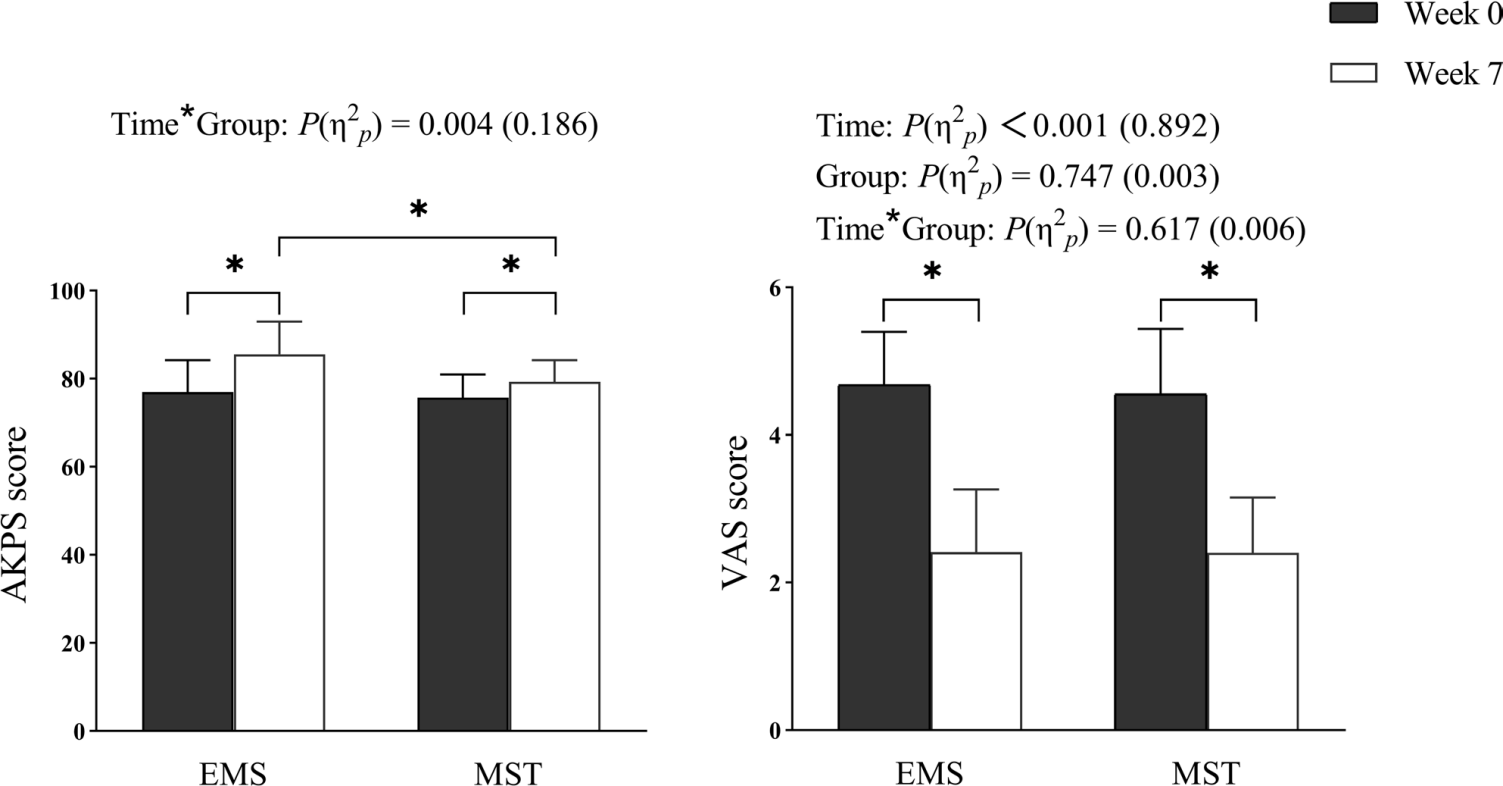
Comparison of AKPS and VAS scores between EMS and MST groups. EMS, electrical muscle stimulation; MST, muscle strength training; AKPS, anterior knee pain scale; VAS, visual analog scale; *P ＜ 0.05

A significant time × group interaction was found for the AKPS scores (*P* = 0.004, η2 *p* = 0.186). Post-hoc tests showed significant improvements in AKPS scores in both the EMS (*P* < 0.001) and MST (*P* = 0.004) groups compared to week 0, with the EMS group demonstrated greater improvement than the MST group at week 7 (*P* = 0.003). For the VAS, a significant main effect of time was observed (*P* < 0.001, η2 *p* = 0.892), with both groups exhibited a significant reduction in VAS scores at week 7 compared to week 0. (Fig. 4)

### Peak torques and muscle activation

A significant time × group interaction was detected for the relative peak torque of knee extension (*P* = 0.028, η2 *p* = 0.118). Post-hoc comparisons indicated a significant increase in the relative peak torque of knee extension in both the EMS (*P* < 0.001) and the MST (*P* = 0.009) group from week 0, with the EMS group showing a greater increase by week 7 (*P* = 0.031). For the relative peak torque of knee flexion (*P* = 0.648, η2 *p* = 0.006), hip extension (*P* = 0.854, η2 *p* = 0.001), hip abduction (*P* = 0.268, η2 *p* = 0.045), and hip external rotation (*P* = 0.122, η2 *p* = 0.086), significant main effects of time were observed. Both groups exhibited significant increases in these parameters at week 7 compared to week 0. (Table 3)

A significant time × group interaction was found for the activation ratio of the vastus medialis and lateralis (*P* = 0.047, η2 *p* = 0.129). Post-hoc comparisons demonstrated that the VM/VL activation ratio in the EMS group was significantly increased compared with week 0 (*P* < 0.001) and was greater than in the MST group at week 7 (*P* = 0.036). Significant main effects of time were observed for muscle activation of the VM (*P* = 0.007, η2 *p* = 0.224), VL (*P* = 0.008, η2 *p* = 0.216), gluteus medius (*P* = 0.001, η2 *p* = 0.254), and gluteus maximus (*P* < 0.001, η2 *p* = 0.399). Compared to week 0, VM activation significantly increased in both groups at week 7, while muscle activation of the VL, gluteus medius, and gluteus maximus significantly decreased. (Table 3)

**Table 3:** Comparison of relative peak torques and muscle activation between EMS and MST groups

Measured variables	Time	EMS group	MST group	Time	Group	Time*Group
				*P* (η2 *p*)	*P* (η2 *p*)	*P* (η2 *p*)
Relative peak torques (Nm·kg^− 1^)						
Knee flexion	Week 0	0.877 ± 0.561	0.871 ± 0.664	<0.001 (0.322)	0.743 (0.003)	0.648 (0.006)
	Week 7	1.310 ± 0.512^a^	1.408 ± 0.520^a^			
Knee extension	Week 0	2.154 ± 0.584	2.242 ± 0.697	-	-	0.028 (0.118)
	Week 7	3.029 ± 0.468 ^ab^	2.653 ± 0.606^a^			
Hip extension	Week 0	2.126 ± 1.175	2.152 ± 0.888	<0.001 (0.450)	0.871 (0.001)	0.854 (0.001)
	Week 7	2.884 ± 1.131^a^	2.973 ± 0.863 ^a^			
Hip abduction	Week 0	0.996 ± 0.333	1.156 ± 0.548	0.021 (0.183)	0.731 (0.004)	0.268 (0.045)
	Week 7	1.386 ± 0.442^a^	1.300 ± 0.261^a^			
Hip external rotation	Week 0	0.415 ± 0.164	0.440 ± 0.142	<0.001 (0.686)	0.131 (0.082)	0.122 (0.086)
	Week 7	0.757 ± 0.320^a^	0.962 ± 0.310^a^			
Muscle activation						
VM/(%)	Week 0	37.5 ± 11.0	37.2 ± 9.6	0.007 (0.224)	0.519 (0.014)	0.345 (0.031)
	Week 7	47.6 ± 14.0^a^	42.3 ± 19.0^a^			
VL/(%)	Week 0	49.6 ± 11.9	49.4 ± 20.3	0.008 (0.216)	0.851 (0.001)	0.788 (0.003)
	Week 7	38.2 ± 12.4^a^	40.1 ± 19.3^a^			
VM/VL (ratio)	Week 0	0.80 ± 0.25	0.90 ± 0.48	-	-	0.047 (0.129)
	Week 7	1.30 ± 0.33^ab^	1.07 ± 0.22			
Gluteus medius/(%)	Week 0	13.1 ± 7.7	14.5 ± 9.2	0.001 (0.254)	0.521 (0.011)	0.792 (0.002)
	Week 7	9.0 ± 3.1^a^	9.7 ± 4.5^a^			
Gluteus maximus/(%)	Week 0	11.4 ± 6.6	13.8 ± 6.6	<0.001 (0.399)	0.375 (0.023)	0.328 (0.027)
	Week 7	8.1 ± 4.4^a^	8.7 ± 4.9^a^			

EMS, electrical muscle stimulation; MST, muscle strength training; VM, vastus medialis; VL, vastus lateralis

^a^Significant difference compared with week 0 (*P* < 0.05), ^b^Significant difference compared with the MST group (*P* < 0.05)

### Hip and knee joint Biomechanical parameters

Significant group × time interactions were detected in the knee external rotation angle (*P* = 0.041, η2 *p* = 0.101), hip adduction angle (*P* = 0.048, η2 *p* = 0.116). Post hoc comparisons indicated that both groups were significantly decreased in the peak knee external rotation angle (EMS: *P* < 0.001; MST: *P* = 0.041) and the peak hip adduction angle (EMS: *P* < 0.001; MST: *P* = 0.004) compared with week 0. The peak knee external rotation angle (*P* = 0.029) and peak hip adduction angle (*P* = 0.003) was lower in the EMS group compared with those in the MST group at week 7. Significant main effects of time were observed on the peak hip abduction (*P* = 0.043, η2 *p* = 0.752) and internal rotation (*P* = 0.039, η2 *p* = 0.116) angles. Both groups exhibited significant reductions in these parameters at week 7 compared to week 0. Significant main effects of time were observed for the peak moments in three anatomical planes at both the knee and hip. Compared to week 0, both groups showed significant reductions in the peak knee extension moment (*P* = 0.033, η2 *p* = 0.111), peak knee abduction moment (*P* = 0.036, η2 *p* = 0.105), peak knee external rotation moment (*P* = 0.002, η2 *p* = 0.216), as well as peak hip extension moment (*P* = 0.017, η2 *p* = 0.141), peak hip abduction moment (*P* = 0.047, η2 *p* = 0.105), and peak hip external rotation moment (*P* = 0.048, η2 *p* = 0.097) at week 7. Additionally, significant main effects of time were observed for both PFJ stress (*P* = 0.045, η2 *p* = 0.102) and F_PFJ_ (*P* = 0.029, η2 *p* = 0.145). Both groups exhibited significant reductions in PFJ stress and F_PFJ_ at week 7 compared to week 0. (Table 4)

**Table 4:** Comparison of hip and knee joint Biomechanical parameters between EMS and MST groups

Measured variables	Time	EMS group	MST group	Time	Group	Time*Group
				*P* (η2 *p*)	*P* (η2 *p*)	*P* (η2 *p*)
Joint angle (°)						
Knee flexion	Week 0	93.0 ± 5.1	90.7 ± 5.6	0.263 (0.031)	0.093 (0.069)	0.776 (0.002)
	Week 7	91.5 ± 4.9	89.8 ± 4.9			
Knee abduction	Week 0	−3.6 ± 5.1	−3.6 ± 6.1	0.043 (0.752)	0.457 (0.203)	0.582 (0.216)
	Week 7	−1.0 ± 2.9^a^	−2.0 ± 6.4^a^			
Knee external rotation	Week 0	−15.7 ± 9.6	−14.0 ± 5.5	-	-	0.041 (0.101)
	Week 7	−5.1 ± 5.8^ab^	−9.6 ± 7.0^a^			
Hip flexion	Week 0	36.9 ± 7.5	38.0 ± 9.4	0.124 (0.058)	0.613 (0.006)	0.904 (<0.001)
	Week 7	34.9 ± 5.6	35.6 ± 5.9			
Hip adduction	Week 0	7.7 ± 2.8	7.4 ± 2.7	-	-	0.048 (0.116)
	Week 7	3.2 ± 2.7^ab^	5.5 ± 3.6^a^			
Hip internal rotation	Week 0	9.2 ± 14.0	9.4 ± 5.6	0.039 (0.116)	0.968 (<0.001)	0.891 (0.001)
	Week 7	4.5 ± 6.8^a^	4.1 ± 9.6^a^			
Joint moment (Nm·kg⁻¹)						
Knee extension	Week 0	−0.944 ± 0.445	−0.955 ± 0.282	0.033 (0.111)	0.736 (0.003)	0.786 (0.002)
	Week 7	−0.768 ± 0.356^a^	−0.817 ± 0.340^a^			
Knee abduction	Week 0	−0.569 ± 0.314	−0.563 ± 0.289	0.036 (0.105)	0.753 (0.003)	0.637 (0.006)
	Week 7	−0.424 ± 0.220^a^	−0.470 ± 0.231^a^			
Knee external rotation	Week 0	−0.110 ± 0.073	−0.098 ± 0.062	0.002 (0.216)	0.606 (0.007)	0.797 (0.002)
	Week 7	−0.073 ± 0.043^a^	−0.066 ± 0.079^a^			
Hip extension	Week 0	−1.013 ± 0.640	−1.164 ± 0.685	0.017 (0.141)	0.508 (0.012)	0.731 (0.003)
	Week 7	−0.799 ± 0.378^a^	−0.881 ± 0.764^a^			
Hip abduction	Week 0	−1.075 ± 0.241	−1.070 ± 0.276	0.047 (0.105)	0.825 (0.001)	0.472 (0.014)
	Week 7	−0.981 ± 0.234^a^	−1.023 ± 0.251^a^			
Hip external rotation	Week 0	−0.416 ± 0.196	−0.419 ± 0.216	0.048 (0.097)	0.705 (0.004)	0.603 (0.007)
	Week 7	−0.345 ± 0.105^a^	−0.377 ± 0.162^a^			
Patellofemoral joint loading						
Peak PFJ stress (MPa·kg⁻¹)	Week 0	0.029 ± 0.012	0.029 ± 0.007	0.045 (0.102)	0.480 (0.013)	0.381 (0.020)
	Week 7	0.021 ± 0.013^a^	0.026 ± 0.016^a^			
Peak F_PFJ_ (N·kg⁻¹)	Week 0	5.834 ± 2.835	5.300 ± 1.720	0.029 (0.145)	0.957 (<0.001)	0.243 (0.044)
	Week 7	4.831 ± 1.974^a^	4.840 ± 2.407^a^			

EMS, electrical muscle stimulation; MST, muscle strength training; PFJ, patellofemoral joint; F_PFJ_, patellofemoral joint reaction force

^a^Significant difference compared with week 0 (*P* < 0.05); ^b^Significant difference compared with the MST group (*P* < 0.05)

## Discussion

This study aimed to investigate the effects of conventional strength training combined with EMS on knee pain, lower-limb muscle strength, and hip/knee joint movement patterns and muscle activation during stair descent in patients with PFP. Our findings demonstrated that patients receiving both intervention programs exhibited improvements in pain, muscle strength, and biomechanical parameters of the hip and knee joints during stair descent. Furthermore, the combined EMS approach produced greater benefits than strength training alone in enhancing AKPS scores, increasing knee extension strength, reducing the peak knee external rotation angle, and decreasing the peak hip adduction angle. In addition, EMS more effectively regulated the activation of the VM and VL muscles.

In the present study, both groups demonstrated a reduction in VAS scores and significant improvements in AKPS scores, muscle strength, and VM activation after six weeks of intervention. Our findings indicate that both intervention protocols were effective in alleviating pain and enhancing knee joint function, which is consistent with previous research [32, 34]. Intriguingly, we observed that the EMS group exhibited higher post-intervention AKPS scores, knee extension strength, and VM/VL activation ratio compared with the MST group. These results suggest that EMS combined with strength training may be more effective in reducing pain, improving knee function, and optimizing the balance of medial and lateral quadriceps activation in patients with PFP. Pain is a common symptom in patients with PFP, affecting muscle function and movement patterns [50, 51]. Greuel et al. reported that knee pain might induce arthrogenic muscle inhibition, resulting in quadriceps weakness and dysfunction [51]. EMS has gained broad clinical application in musculoskeletal rehabilitation, as it not only alleviates pain but also promotes selective activation of target muscles by recruiting small motor units [23]. A meta-analysis on neuromuscular electrical stimulation after ACL reconstruction demonstrated that incorporating EMS significantly enhanced quadriceps strength in the early postoperative period [24]. Accordingly, the greater benefits in the EMS group are likely attributable to the pain-inhibitory response elicited by EMS, which reduces pain and may enhance motor unit recruitment.

Although, contrary to expectations, activation of the gluteus medius and gluteus maximus during stair descent was reduced in patients with PFP after the 6-week intervention, we nevertheless observed a significant improvement in hip muscle strength. Similar to quadriceps inhibition, hip muscle activation may also be inhibited in patients with PFP [52]. The reduction in gluteal muscle activation may represent a compensatory strategy adopted by patients with PFP during functional activities as a result of chronic pain [16]. Previous research indicates that knee injuries may arise from proximal muscle dysfunction, particularly hip muscles weakness, which impairs femoral control [53]. This dysfunction increases dynamic knee valgus, elevating friction between the posterior patellar cartilage and the lateral femoral condyle, potentially inducing inflammation and pain [54]. Furthermore, the gluteus medius stabilizes the pelvis and femoral head, while the gluteus maximus provides three-dimensional stability against hip flexion, adduction, and internal rotation [55]. Thus, controlling femoral movements is critical for restoring normal patellofemoral joint loading. Notably, we also found that patellofemoral joint stress, reaction force, and dynamic knee valgus (as elaborated in the following section) during stair descent were significantly reduced in both groups after the intervention. This improvement was most likely attributable to the intervention-induced enhancement of gluteal muscle strength in patients with PFP.

The present study found that dynamic knee valgus during stair descent was significantly diminished in both groups following the intervention. This indicates that the 6-week intervention may decrease the risk of PFP. Additionally, compared to the MST group, the EMS group exhibited a more significant reduction in knee external rotation and hip adduction angles. This suggests that incorporating EMS may provide a superior therapeutic effect for PFP. Dynamic knee valgus constitutes an abnormal lower-limb movement pattern. It is influenced by a complex movement pattern, including hip adduction and internal rotation, as well as knee abduction and external rotation [54]. It has been reported that approximately 66% of patients with PFP exhibit dynamic knee valgus during functional activities, and that dynamic knee valgus is positively associated with pain [56, 57]. This indicates that elevated dynamic knee valgus may be one of the primary risk factors for PFP and is considered a reliable approach for identifying lower-limb injuries, including PFP [58–60]. Related studies demonstrated that PFP patient exhibited greater knee external rotation and hip adduction during various functional activities [61]. Li et al. found that elevated tibial external rotation might have diminished joint contact area [62]. This resulted in a 10–24% elevation in PFJ stress. The lateral tilt and displacement of the patella during weight-bearing activities arose from excessive femoral adduction and internal rotation, rather than patellar movement [63]. Therefore, improving the multi-plane movement patterns of the hip and knee joints was essential for diminishing patellofemoral joint contact force. Notably, our study demonstrated that incorporating EMS provided greater benefits in reducing knee external rotation and hip adduction angles. This may be attributed to EMS selectively activating the VM, which improved joint-derived muscle inhibition, alleviated abnormal neuromuscular activity, and enhanced neuromuscular control of the lower limb.

In the present study, VM activation did not differ significantly between the two groups after the intervention, consistent with a meta-analysis indicating that adding EMS to exercise programs is not superior to conventional physiotherapy in improving muscle activation [64]. Furthermore, this study demonstrated no significant difference in patellofemoral joint loading between the two groups following the intervention. This finding may be related to the relatively short duration of the intervention in this study, which might have limited our ability to detect any potential superior efficacy of the combined intervention. Although the between-group difference did not reach statistical significance, both peak PFJ stress and FPFJ were lower in the EMS group than in the MST group at post-intervention. In summary, the incorporation of EMS demonstrated more pronounced training effects in diminishing dynamic knee valgus, enhancing knee extension strength, and alleviating pain. However, in certain measures, the effects of EMS could not be distinguished from the positive effects of strength training alone.

There are some limitations to this study. Firstly, we did not examine the biomechanical characteristics of the trunk, pelvis, or ankle joint. However, evidence indicates that trunk flexion, pelvic tilt, and ankle joint movement may influence knee joint loading during stair descent [43]. Secondly, differences in movement patterns, pain presentation, and anatomical structure between male and female patients may affect the etiologic triggers, symptoms, and rehabilitation strategies for PFP [65, 66]. The effect of gender should also be investigated in future studies to facilitate the development of more targeted treatment programs. In addition, findings from this study should be cautiously applied to other functional screening tasks.

## Conclusions

EMS combined with strength training more effectively mitigated abnormal hip-knee movement patterns during stair descent, balanced the activation of the vastus medialis and lateralis, increased knee extensor strength, and alleviated pain and enhanced function in PFP patients. Therefore, adding EMS to muscle training may be recommended as one of the clinical treatments for individuals with PFP.

## Supplementary Information

Supplementary Material 1.

## Data Availability

The data used to support the findings of this study are available from the corresponding author upon reasonable request.

## Abbreviations

PFP: Patellofemoral pain
EMS: Electrical muscle stimulation
MST: Muscle strength training
EMG: Electromyography
VM: Vastus medialis
VL: Vastus lateralis
ACL: Anterior cruciate ligament
VAS: Visual analog scale
AKPS: Anterior knee pain scale
RM: Repetition maximum
LB: Pound
MVIC: Maximum voluntary isometric contraction
GRF: Ground reaction force
PFJ: Patellofemoral joint
F_PFJ_: Patellofemoral joint reaction force
